# Supplementary material for: Undernutrition Scored Using the CONUT Score with Hypoglycemic Status in ICU-Admitted Elderly Patients with Sepsis Shows Increased ICU Mortality
Source: Diagnostics (Basel). 2023 Feb 17;13(4):762. doi: 10.3390/diagnostics13040762 (PMC9955230; doi:10.3390/diagnostics13040762)
Supplement: Supplementary file 1 [file diagnostics-13-00762-s001.zip › TableS1_230206JY.pdf]

Supplementary Table S1. Assessment of the undernutrition status using the CONUT score*

	Undernutrition Status			
**Parameter**	**Normal**	**Light**	**Moderate**	**Severe**
Serum Albumin (g/dL)	3.5–4.5	3.0–3.49	2.5–2.9	<2.5
Alb Score	**0**	**2**	**4**	**6**
Total Lymphocytes/μL	>1600	1200–1599	800–1199	<800
TLC Score	**0**	**1**	**2**	**3**
Total Cholesterol (mg/dL)	>180	140–180	100–139	<100
T-cho Score	0	1	2	3
Screening Total Score	**0–1**	**2–4**	**5–8**	**9–12**

* The CONUT score = (Alb Score) + (TLC Score) + (T-cho score)

Abbreviations: Alb, Serum Albumin; TLC, Total lymphocytes; T-cho, Total cholesterol
