# Supplementary material for: Undernutrition Scored Using the CONUT Score with Hypoglycemic Status in ICU-Admitted Elderly Patients with Sepsis Shows Increased ICU Mortality
Source: Diagnostics (Basel). 2023 Feb 17;13(4):762. doi: 10.3390/diagnostics13040762 (PMC9955230; doi:10.3390/diagnostics13040762)
Supplement: Supplementary file 1 [file diagnostics-13-00762-s001.zip › TableS2-230206JY.pdf]

Supplementary Table S2. Origin of infection in each glycemic group

Origin of infection	Hypo-G (n = 46)	Inter-G (n = 183)	Hyper-G (n = 75)
Lung (n = 135)	13/46 (28.3%)	91/183 (49.7%)	31/75(41.3%)
Urine tract (n = 64)	9/46 (19.6%)	40/183 (21.9%)	15/75 (20.0%)
Abdomen (n = 46)	9/46 (19.6%)	22/183 (12.0%)	15/75 (20.0%)
Soft tissue (n = 20)	7/46 (15.2%)	10/183 (5.5%)	2/75 (2.7%)
Central Nervous System (n = 6)	0/46 (0%)	4/183 (2.2%)	2/75 (2.7%)
Endocardial systems (n = 3)	0/46 (0%)	2/183 (1.1%)	1/75 (1.3%)
Others (n = 33)	8/46(17.4%)	14/183 (7.7%)	9/75 (12.0%)

Abbreviations: Hypo-G, Hypoglycemia group (blood glucose level <80 mg/dL); Hyper-G, hyperglycemia group (blood glucose level ≧200 mg/dL); Inter-G, intermediate glycemia group (blood glucose level = 80–119 mg/dL); Others, including cases of unknown origin
