# Supplementary material for: Undernutrition Scored Using the CONUT Score with Hypoglycemic Status in ICU-Admitted Elderly Patients with Sepsis Shows Increased ICU Mortality
Source: Diagnostics (Basel). 2023 Feb 17;13(4):762. doi: 10.3390/diagnostics13040762 (PMC9955230; doi:10.3390/diagnostics13040762)
Supplement: Supplementary file 1 [file diagnostics-13-00762-s001.zip › TableS3-230206JY.pdf]

Supplementary Table S3. Comparison of causative pathogens among groups (n=307)

Causative pathogen	All	Survivors	Non-survivors	P-value *
**Gram-negative bacteria**	118	95/118	23/118	0.724
**Gram-positive bacteria**	87	67/87	20/87	0.501
**Fungi**	7	5/7	2/7	0.594
**None**	46	38/46	8/46	0.569
**Non-specific**	29	39/49	10/49	0.983

Abbreviations: None, No bacteria were detected in the culture; Non-specific, Normal flora or multiple bacteria were detected but no causative pathogenic bacteria were identified.

*Chi-square tests were performed for analysis between survivors and non-survivors.
