# Supplementary material for: Undernutrition Scored Using the CONUT Score with Hypoglycemic Status in ICU-Admitted Elderly Patients with Sepsis Shows Increased ICU Mortality
Source: Diagnostics (Basel). 2023 Feb 17;13(4):762. doi: 10.3390/diagnostics13040762 (PMC9955230; doi:10.3390/diagnostics13040762)
Supplement: Supplementary file 1 [file diagnostics-13-00762-s001.zip › TableS4-230206JY.pdf]

Supplementary Table S4. Comparisons by causative pathogens (n=307)

Causative pathogen	Gram-negative bacteria (n=118)	Gram-positive bacteria (n=87)	Fungi (n=7)	None (n=46)	Non-specific (n=29)	P-value*
**CONUT score****	7.18±3.03	7.54±3.28	6.17±2.23	5.41±3.35	6.45±3.17	0.029
**BG (mg/ml)**	169.8±168.2	159.4±128.1	167.9±56.5	212.5±247	184.2±79.4	0.245
**Ratio of septic shock (%)**	63/117	43/84	2/7	15/45	29/48	0.059
**Ratio of Hypo-G (%)**	23/116	15/86	0/7	6/46	2/49	0.078
**Ratio of Inter-G (%)**	69/116	53/86	5/7	26/46	30/49	0.945
**Ratio of Hyper-G (%)**	24/116	18/86	2/7	14/46	17/49	0.271
**SOFA score**	8.61±3.57	8.03±2.94	6.71±2.99	7.54±3.25	7.73±2.99	0.844

Abbreviations: CONUT score, Controlling Nutritional Status score; BG, Blood Glucose; Ratio of septic shock, Ratio of septic shock/ Total number of patients; Hypo-G, Hypoglycemia group (blood glucose level <80 mg/dL); Hyper-G, hyperglycemia group (blood glucose level ≧ 200 mg/dL); Inter-G, intermediate glycemia group (blood glucose level = 80–119 mg/dL);
None, No bacteria were detected in the culture. Non-specific, Normal flora or multiple bacteria were detected but no causative pathogenic bacteria were identified.

SOFA score obtained at admission (mean±SD)
*Kruskal-Wallis test was conducted for multiple comparisons among CONUT scores, BG, and SOFA scores, and the origin of infection.
**Chi-square tests were performed to analyze the ratio of septic shock in each of the three glycemic groups (Hypo-G, Inter-G, Hyper-G). We determined the optimal cut-off and the significance level was 5%.
**Bonferroni test was conducted
